# Deletions in the Repertoire of *Pseudomonas syringae* pv. *tomato* DC3000 Type III Secretion Effector Genes Reveal Functional Overlap among Effectors

**DOI:** 10.1371/journal.ppat.1000388

**Published:** 2009-04-17

**Authors:** Brian H. Kvitko, Duck Hwan Park, André C. Velásquez, Chia-Fong Wei, Alistair B. Russell, Gregory B. Martin, David J. Schneider, Alan Collmer

**Affiliations:** 1 Department of Plant Pathology and Plant-Microbe Biology, Cornell University, Ithaca, New York, United States of America; 2 Boyce Thompson Institute for Plant Research, Ithaca, New York, United States of America; 3 United States Department of Agriculture–Agricultural Research Service, Robert W. Holley Center for Agriculture and Health, Ithaca, New York, United States of America; The University of North Carolina at Chapel Hill, United States of America

## Abstract

The γ-proteobacterial plant pathogen *Pseudomonas syringae* pv. *tomato* DC3000 uses the type III secretion system to inject ca. 28 Avr/Hop effector proteins into plants, which enables the bacterium to grow from low inoculum levels to produce bacterial speck symptoms in tomato, *Arabidopsis thaliana*, and (when lacking *hopQ1-1*) *Nicotiana benthamiana.* The effectors are collectively essential but individually dispensable for the ability of the bacteria to defeat defenses, grow, and produce symptoms in plants. Eighteen of the effector genes are clustered in six genomic islands/islets. Combinatorial deletions involving these clusters and two of the remaining effector genes revealed a redundancy-based structure in the effector repertoire, such that some deletions diminished growth in *N. benthamiana* only in combination with other deletions. Much of the ability of DC3000 to grow in *N. benthamiana* was found to be due to five effectors in two redundant-effector groups (REGs), which appear to separately target two high-level processes in plant defense: perception of external pathogen signals (AvrPto and AvrPtoB) and deployment of antimicrobial factors (AvrE, HopM1, HopR1). Further support for the membership of HopR1 in the same REG as AvrE was gained through bioinformatic analysis, revealing the existence of an AvrE/DspA/E/HopR effector superfamily, which has representatives in virtually all groups of proteobacterial plant pathogens that deploy type III effectors.

## Author Summary

*Pseudomonas syringae* is a Gram-negative plant pathogen that defeats plant defenses through effector proteins that are injected into plant cells via the type III secretion system. *P. syringae* strains are assigned to pathovars based largely on their host of origin. *P. syringae* pv. *tomato* DC3000 causes bacterial speck of tomato and has become a model for studying bacterium–plant interactions because it also attacks the experimentally amenable plants *Arabidopsis thaliana* and (if one effector acting as an avirulence determinant is removed) *Nicotiana benthamiana*. Genome sequence–enabled studies have revealed that strains in different pathovars harbor large (15–30) effector repertoires, which are surprisingly diverse and show no obvious correlation with host range. In search of rules governing the composition of effector repertoires in individual strains, we constructed combinatorial deletions involving 20 of the 28 effectors deployed by D3000. The pattern of growth defects resulting from these mutations suggests an architecture in the effector repertoire involving redundant targeting of a few vulnerable plant defense processes and compensatory redundancies in these defenses.

## Introduction

Many bacterial pathogens of plants and animals disarm and remodel host cells by injecting large repertoires of effectors via the type III secretion system (T3SS) [1],[2]. In some cases, the repertoires of individual strains appear to function as robust systems that can tolerate loss of individual effectors with little or no reduction in virulence because of high-level functional overlap among the effectors [3]. The potential extent of such redundancy is highlighted by recent reports that enterohemorrhagic *Escherichia coli* 0157:H7 may inject 39 effectors into animal cells via the T3SS and that fungal and oomycete pathogens, using alternative protein translocation pathways, may deliver hundreds of effectors into plant cells [4]–[7]. Unraveling functional redundancy among effectors can potentiate the study of individual effectors through the design of mutants with more easily assayed phenotypes, and elucidation of functional overlaps should help us understand how the various effectors in a repertoire may function as a system in hosts.

*Pseudomonas syringae* pv. *tomato* DC3000, which causes bacterial speck of tomato, is an excellent model for investigating the possible operation of type III effector repertoires as systems. The DC3000 genome has been fully sequenced [8]; the DC3000 T3SS, which is encoded by *hrp*/*hrc* (hypersensitive response and pathogenicity or conserved) genes, is being intensively investigated [9]–[12]; multiple approaches have been used to firmly establish the effector repertoire [13]–[15]; the molecular function of several of these effectors in plants has been determined [16]–[18]; DC3000 can infect the experimentally tractable plants *Arabidopsis thaliana* and *Nicotiana benthamiana* [19],[20]; and this strain has become the pathogen of choice for plant biologists probing the plant defense systems thought to be targeted by the effectors. 

DC3000 appears to actively deploy 28 effectors and several other proteins associated with extracellular functions of the T3SS [14],[21]. The genome also harbors 12 effector pseudogenes and seven effector genes that appear only weakly expressed [14],[21],[22]. The majority of the active effector genes occur within six clusters in the DC3000 genome [23]. By deleting various effector gene clusters and individual genes, we have shown that effector HopQ1-1 (Hop denotes Hrp outer protein) acts as an avirulence determinant in *N. benthamiana*, that deleting *hopQ1-1* enables DC3000 to cause bacterial speck disease in *N. benthamiana* (which is otherwise a nonhost), and that deleting four of the clusters (encoding 12 effectors) strongly reduces symptom production in *N. benthamiana* but surprisingly does not significantly reduce bacterial growth [20].

According to the current model for *P. syringae*-plant interactions [24], the primary function of the effectors is to suppress PAMP (pathogen-associated molecular pattern)-triggered immunity (PTI), which is elicited by common bacterial factors like flagellin, LPS, peptidoglycan, and elongation factor Tu. PAMPs are perceived by pattern recognition receptors (receptor-like kinases) at the surface of plant cells. A second layer of defense involves detection inside plant cells of injected effectors or their activity by resistance (R) proteins, which results in effector-triggered immunity (ETI). Pathogens may evade ETI by mutating the betraying effector gene or by deploying another effector that suppresses ETI. This model predicts a coevolutionary process that would generate the observed amplification of effector genes in pathogens and of *R* genes in plants, and the model also predicts interplay among effectors in redundantly targeting PTI and suppressing ETI.

Three observations support the prediction of interplay among effectors in a repertoire. In the first example, loss of *virPphA* (*hopAB1*) from *P. syringae* pv. *phaseolicola* 1449B resulted in avirulence in bean, the normal host, because of failure to suppress ETI triggered by another effector in the 1449B repertoire [25]. The other two examples of effector interplay involve work with DC3000 in host tomato and provide a foundation for the work presented here. Although mutations in individual *P. syringae* effector genes typically produce little or no loss in growth in planta, an Δ*avrE*Δ*hopM1* double mutant and an Δ*avrPto*Δ*avrPtoB* double mutant were found to be significantly reduced in growth in tomato [26],[27]. The *avrPto* and *avrPtoB* genes are unlinked in the DC3000 genome, but the observation that these two effectors make redundant contributions to virulence is consistent with the observation that they target the FLS2/BAK1 pattern recognition receptor complex important in PAMP detection [28],[29].

The *avrE* and *hopM1* genes are located in DC3000 effector cluster VI [23], which is known as the conserved effector locus (CEL) [30]. The CEL forms part of the tripartite Hrp pathogenicity island of *P. syringae*, and *avrE*, *hopM1*, and *hopAA1-1* are present in this location in diverse *P. syringae* strains [13]. We had previously shown that deletion of the CEL region encompassing *avrE*, *hopM1*, and *hopAA1-1* strongly reduces DC3000 virulence [30]. AvrE and HopM1 appear to play a major role in promoting cell death in both host and nonhost plants. This ability is not attributable to avirulence activity, as indicated by experiments involving host tomato and nonhost tobacco (*Nicotiana tabacum*) challenged with DC3000 mutants lacking both effectors or with *Agrobacterium*-mediated transient expression of AvrE in these plants [26],[31]. Importantly, the ΔCEL mutant fails to suppress PTI-associated callose deposition in Arabidopsis, and either *avrE* or *hopM1* can redundantly restore callose suppression and bacterial growth to the ΔCEL mutant [32].

Here, we extend our analysis of DC3000 effector gene cluster polymutants by deleting cluster I and the CEL to produce a strain lacking all 18 of the clustered effector genes. We explore the basis for the failure of the resulting mutant to grow well in *N. benthamiana*, and in so doing we identify effector gene clusters that functionally overlap with the CEL. This analysis leads to HopR1 in cluster IV, which we show to be a member of an effector superfamily that also contains the AvrE family and which also contributes to the suppression of PTI-associated callose deposition in *N. benthamiana* leaves inoculated with DC3000. We then explore the effects of deleting the flagellin PAMP-encoding *fliC* gene on the growth of DC3000 effector polymutants. Our observations lead to the proposal that AvrE/HopR1/HopM1 and AvrPto/AvrPtoB form redundant-effector groups (REGs) that make major contributions to DC3000 growth in *N. benthamiana* by targeting different steps in PTI.

## Results

### The construction of *P. syringae* pv. *tomato* DC3000 mutants with deletions involving all of the clustered effector genes reveals the relative importance of the CEL in bacterial growth in *N. benthamiana* and tomato

**Figure 1:**
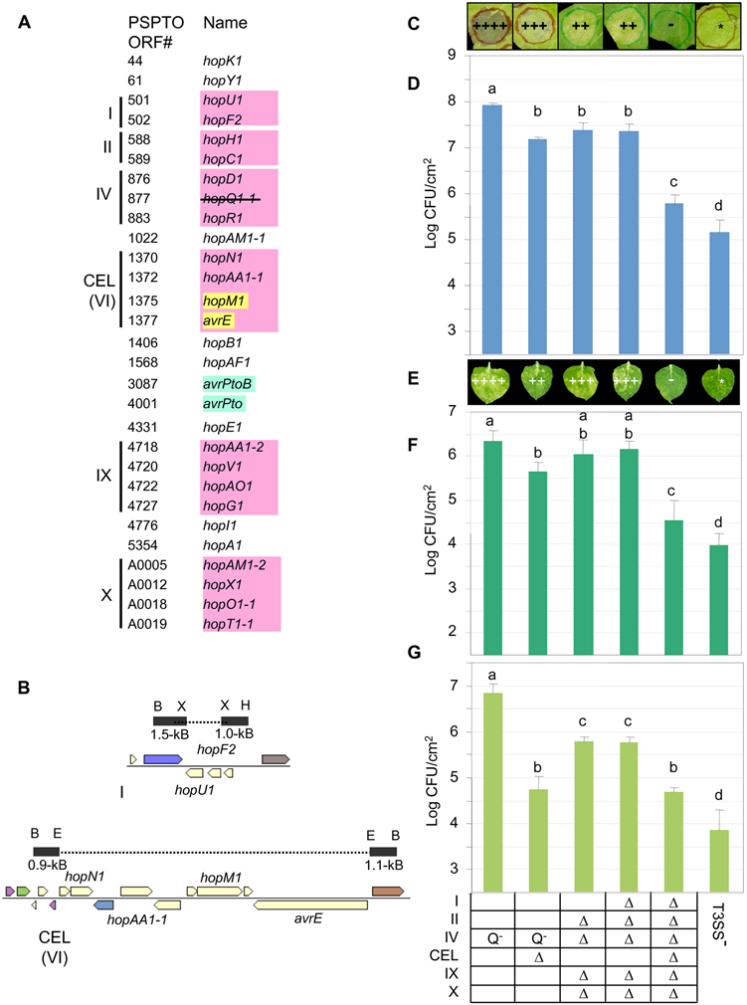
Polymutants lacking clustered effector genes highlight the importance of the CEL in virulence. (A) The *P. syringae* pv. *tomato* DC3000 genome harbors 18 active effectors in six clusters (shaded pink), including the conserved effector locus (CEL). Cluster X is carried on pDC3000A and ΔX strains have been cured of the plasmid. Additional clusters harboring apparently inactive effector genes and pseudogenes are not shown. The *hopM1* and *avrE* genes are shaded yellow to denote that they functionally overlap; the *avrPto* and *avrPtoB* genes are similarly shaded blue to denote that they are functionally linked although not genetically clustered; and a line is shown through *hopQ1-1* to indicate that all strains used in this study lack this effector because of its avirulence activity in *N. benthamiana*. (B) Effector gene clusters I and CEL(VI) were deleted using pK18*mobsacB*. Flanks were amplified by PCR with terminal primer-introduced restriction sites as labeled. Genes are colored using COG function category defaults. B, *Bam*HI; X, *Xba*I; H, *Hin*dIII; E, *Eco*RI. (C) Deletion of all of the clustered effector genes abolishes the ability of DC3000 to cause symptoms in *N. benthamiana* leaf areas inoculated via a blunt syringe. The marked leaf areas were infiltrated with the strains indicated in the table below at 3×10^4^ CFU/ml and photographed 9 days post-inoculation. ++++, extensive chlorosis and necrosis; +++ extensive chlorosis and reduced necrosis; ++, reduced chlorosis and highly reduced/delayed necrosis; -, no symptoms; *, limited chlorosis no necrosis. “Q^−^” in the table indicates only *hopQ1-1* has been deleted from cluster IV. The T3SS^−^ mutant was Δ*hrcQ_B_-hrcU* CUCPB5113. (D) Deletion of all of the clustered effector genes strongly reduces the ability of DC3000 to grow in *N. benthamiana* leaf areas inoculated via a blunt syringe. *N. benthamiana* leaves were infiltrated with the strains indicated in the table at 3×10^4^ CFU/ml (2.5 log CFU/cm^2^ of leaf tissue). Bacterial populations were determined from three 0.8-cm leaf discs 6 days post-inoculation. Results are the mean and standard deviation of bacterial populations collected from four leaf samples. Means marked with the same letter are not statistically different at the 5% confidence level based on Duncan's multiple range test. This experiment was repeated three times with similar results. (E) The ΔCEL mutant is significantly reduced in symptom production when *N. benthamiana* leaves are inoculated by dipping. *N. benthamiana* leaves were inoculated with the strains indicated in the table at 3×10^5^ CFU/ml and photographed 9 days post-inoculation. ++++, extensive chlorosis and necrotic specks; +++, extensive chlorosis and reduced necrotic specks; ++, reduced chlorosis and reduced necrotic specks; -, no symptoms; *, limited chlorosis and no necrotic specks. (F) Deletion of all of the clustered effector genes strongly reduces the ability of DC3000 to grow in *N. benthamiana* leaves inoculated by dipping. Whole *N. benthamiana* plants were dipped in 3×10^5^ CFU/ml suspensions of the strains indicated in the table (1.5 log CFU/cm^2^ of leaf tissue). Bacterial populations were determined from three 0.8-cm leaf discs 6 days post-inoculation. Results are the mean and standard deviation of bacterial populations collected from four leaf samples. Means marked with the same letter are not statistically different at the 5% confidence level based on Duncan's multiple range test. This experiment was repeated two times with similar results. (G) Deletion of all of the clustered effector genes strongly reduces the ability of DC3000 to grow in tomato leaf areas inoculated via a blunt syringe. Tomato leaflets were infiltrated with the strains indicated in the table at 3×10^4^ CFU/ml (2.5 log CFU/cm^2^ leaf tissue) with a blunt syringe. Bacterial populations were determined from three 0.8-cm leaf discs 3 days post-inoculation. Results are the mean and standard deviation of bacterial populations collected from four leaflet samples. Means marked with the same letter are not statistically different at the 5% confidence level based on Duncan's multiple range test. This experiment was repeated three times with similar results.

As shown in Figure 1A, 18 of the DC3000 active effector genes occur in 6 clusters (with the duplicated *hopAM1-1* and *hopAM1-2* genes counted as just *hopAM1-1* in this tally). We used pK18*mobsacB* as before to construct unmarked deletions involving cluster I and the CEL in various mutants lacking other effector genes (Figure 1B) [20]. We then analyzed the ability of these mutants to grow and produce symptoms in *N. benthamiana* leaves when inoculated by syringe infiltration or dipping and to grow in tomato leaves when inoculated by syringe infiltration (Figure 1 C–1G). With the exception of the Δ*hrcQ_B_-hrcU* (T3SS^−^) strain, all mutants carried deletions affecting *hopQ1-1* or the entire cluster IV, which is necessary to avoid HopQ1-1-mediated avirulence in *N. benthamiana*.

The ΔCEL mutant showed a significant reduction in growth in dip-inoculated *N. benthamiana*, and was strongly reduced in growth in tomato, as was previously observed with Δ*hopAA1*-1Δ*hopM1*Δ*avrE* and Δ*hopM1*Δ*avrE* CEL mutants [26],[30]. Deletion of cluster I in a ΔIIΔIVΔIXΔX background produced no virulence reduction in any of our tests. However, a strong reduction in growth in planta and a complete loss of symptom production was observed when the CEL was deleted from the ΔIΔIIΔIVΔIXΔX background to produce mutant CUCPB5500, which lacks all 18 of the clustered effector genes. A CUCPB5500 derivative was confirmed to perform as well as DC3000 in growth on Hrp-inducing minimal medium and mannitol-glutamate MG medium and in translocation of AvrPto-Cya, expressed from its native promoter in pCPP5702 [11], into *N. benthamiana* when inoculated at 1×10^8^ or 1×10^7^ CFU/ml (Figure S1). Thus, the failure of ΔIΔIIΔIVΔCELΔIXΔX DC3000 to grow well in *N. benthamiana* can be attributed to a subset of the 18 missing effectors that is important in plant interactions.

Several aspects of these observations are notable. First, dipping leaves in inoculum is considered a more natural method of inoculation than infiltration with a blunt syringe and a better test for subtle reductions in virulence. Nonetheless, we saw only minor differences in the relative symptoms in *N. benthamiana* leaves produced by our panel of mutants using the two inoculation methods (Figure 1C and 1E), and the relative growth in the differently inoculated leaves was essentially identical (Figure 1D and 1F). Given the convenience and higher reproducibility of the syringe infiltration assays, we used this method in all subsequent experiments. Second, the CEL was more important for growth in tomato than in *N. benthamiana*. However, the strong reduction in growth in *N. benthamiana* resulting from deleting the CEL from the ΔIΔIIΔIVΔIXΔX mutant revealed the underlying importance of the CEL and suggested that one or more effectors encoded outside of the CEL acts redundantly with CEL effectors. Third, results of virulence and growth assays of the ΔIΔIIΔIVΔCELΔIXΔX mutant suggest that the remaining 10 effectors were not sufficient to promote more than minimally significant growth of the mutant above the T3SS^−^ mutant in all plant tests.

### Analysis of combinatorial deletions reveals effector gene clusters that functionally overlap in enabling bacterial growth and symptom production in *N. benthamiana*

**Figure 2:**
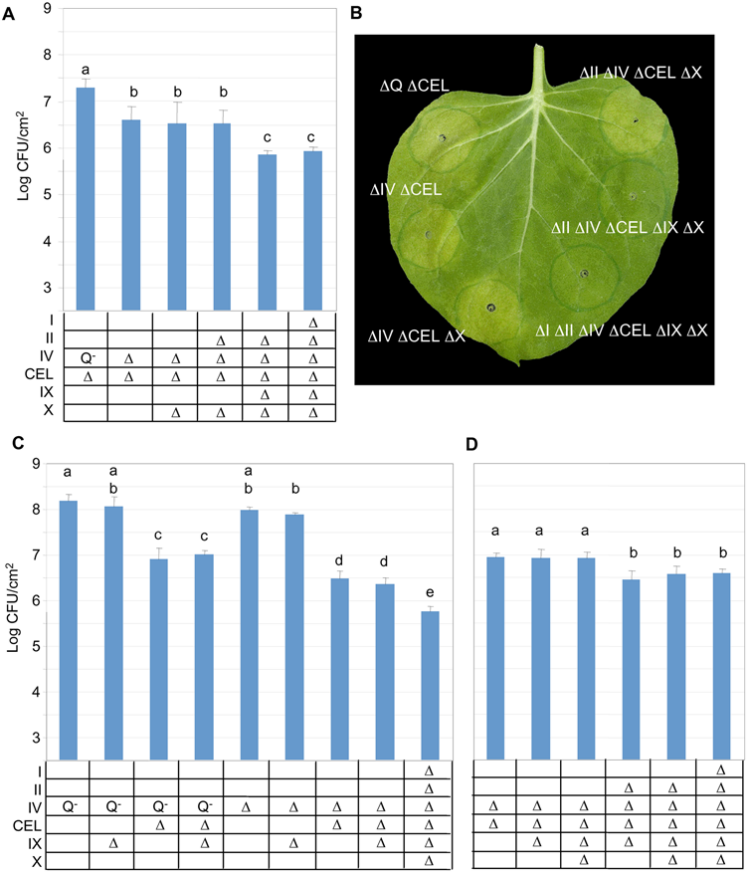
Combinatorial deletions reveal effector gene clusters that interplay in promoting virulence in *N. benthamiana*. (A) Deleting the CEL cluster from intermediates in the lineage of polymutants used to delete all clustered effector genes reveals functional overlap between the CEL and clusters IV and IX in promoting bacterial growth in *N. benthamiana*. Leaves were infiltrated with the strains indicated in the table at 3×10^4^ CFU/ml (2.5 log CFU/cm^2^ leaf tissue) with a blunt syringe. “Q^−^” in the table indicates only *hopQ1-1* has been deleted from cluster IV. Bacterial populations were determined from three 0.8-cm leaf discs 6 days post-inoculation. Results are the mean and standard deviation of bacterial populations collected from four leaf samples. Means marked with the same letter are not statistically different at the 5% confidence level based on Duncan's multiple range test. This experiment was repeated three times with similar results. (B) *N. benthamiana* leaves were infiltrated with the strains indicated at 3×10^4^ CFU/ml with a blunt syringe and photographed 6 days post-inoculation. (C) Additional effector gene cluster polymutants further define functional redundancies with cluster IX in promoting bacterial growth in *N. benthamiana*. Leaves were infiltrated and assayed as described in (A). This experiment was repeated three times with similar results. (D) Additional effector gene cluster polymutants reveal functional overlap between clusters II and IX in promoting bacterial growth in *N. benthamiana*. Leaves were infiltrated and assayed as described in (A). This experiment was repeated three times with similar results.

To identify any effector gene clusters that function redundantly with the CEL we introduced the ΔCEL mutation into the Δ*hopQ1-1* mutant and the lineage of mutants that were used to construct the ΔIΔIIΔIVΔCELΔIXΔX strain. The mutants were inoculated into *N. benthamiana* leaves and assayed for growth 6 days later. As shown in Figure 2A the ΔIVΔCEL mutant was significantly impaired in growth relative to the Δ*hopQ1-1*ΔCEL mutant. Additional deletions of clusters X, II, and I had no significant effect, but deletion of cluster IX from a ΔIIΔIVΔCELΔX mutant resulted in a further reduction in growth. Interestingly, all mutants with the ΔCELΔIX mutation no longer elicited chlorosis in *N. benthamiana* leaves (Figure 2B).

A slight reduction in growth of the ΔIV mutant relative to the Δ*hopQ1-1* mutant was also observed (Figure 2C), however, the reduction was not significant in this experiment and was absent or insignificant in five of eight repeated experiments; in contrast, in all experiments, the ΔIVΔCEL mutant was significantly impaired in growth relative to the Δ*hopQ1-1*ΔCEL mutant (data not shown). This suggests that the ΔIVΔCEL mutant growth impairment does not result from an additive effect of mutations involving cluster IV and the CEL.

The cluster IX deletion was introduced when the lineage already contained multiple effector gene cluster deletions. Therefore, to uncover underlying functional redundancies of other clusters with cluster IX, we constructed pairs of deletion polymutants lacking *hopQ1-1* or cluster IV along with additional clusters, with one member of each pair also lacking cluster IX. This revealed that deleting cluster IX had little impact on the growth of the Δ*hopQ1-1*, ΔCEL, or ΔIVΔCEL mutants (Figure 2C). However, the ΔIΔIIΔIVΔCELΔIXΔX mutant was more strongly reduced in growth than the ΔIVΔCELΔIX mutant, which suggested that cluster II or cluster X was acting redundantly with cluster IX.

We constructed another series of deletions in which every strain carried the ΔIVΔCEL mutations plus additional deletions involving cluster IX and candidate redundant clusters. Deletion of cluster II from the ΔCELΔIVΔIX mutant significantly reduced bacterial growth, and no further reduction was observed upon further deletion of clusters I and X (Figure 2D). Thus, deletion of the CEL is sufficient to reduce bacterial growth in *N. benthamiana*, but the stronger growth reductions observed with polymutants reveal that a cluster IV effector other than HopQ1-1 functions redundantly with the CEL, and in a ΔIVΔCEL background further potential redundancies with clusters II and IX are uncovered.

### Complementation analysis reveals that HopR1 is functionally redundant with the CEL in promoting bacterial growth in *N. benthamiana*, but is less effective in promoting bacterial growth in tomato

**Figure 3:**
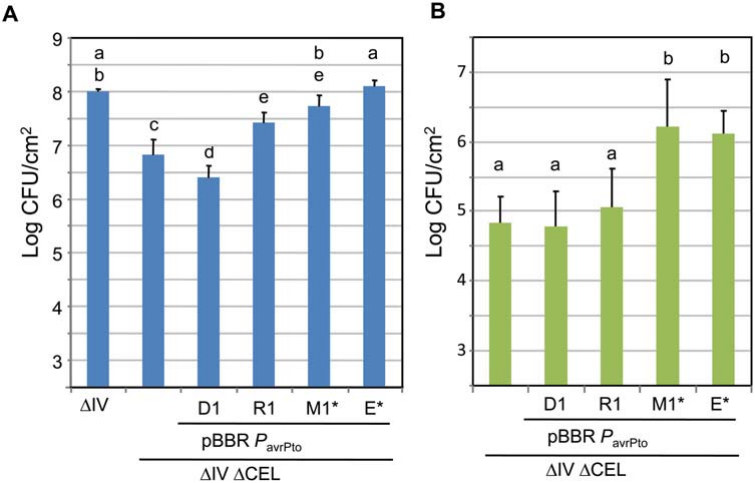
HopR1 is functionally redundant with the CEL in promoting bacterial growth in *N. benthamiana*. (A) *N. benthamiana* leaves were infiltrated with ΔIV or ΔIVΔCEL mutant strains transformed with pBBR *P*_avrPto_ *hop* expression constructs at 3×10^4^ CFU/ml (2.5 log CFU/cm^2^ leaf tissue) with a blunt syringe. The *avr*/*hop* genes carried by the constructs are indicated by the final portion of their gene names. Asterisks indicate that the construct also expresses the appropriate effector chaperone. Bacterial populations were determined from three 0.8-cm leaf discs 6 days post-inoculation. Results are the mean and standard deviation of bacterial populations collected from four leaf samples. Means marked with the same letter are not statistically different at the 5% confidence level based on Duncan's multiple range test. This experiment was repeated four times with similar results. Tomato leaflets were infiltrated with the indicated strains (described further in Table 1) at 3×10^4^ CFU/ml (2.5 log CFU/cm^2^ leaf tissue) with a blunt syringe. Bacterial populations were determined from three 0.8-cm leaf discs 3 days post-inoculation. Results are the mean and standard deviation of bacterial populations collected from three separate experiments with four leaflets each. Means marked with the same letter are not statistically different at the 5% confidence level based on Duncan's multiple range test.

The experiments above suggested that an effector in cluster IV was functionally redundant with CEL effectors. Cluster IV contains *hopD1*, *hopQ1-1*, and *hopR1*. We cloned *hopD1* and *hopR1* and the CEL effector genes *hopM1* and *avrE* (and their respective chaperone genes, *shcM* and *shcE*) under the direction of the *avrPto* promoter in the broad-host-range vector pBBR derivative pCPP5372 [12]. These constructs were then expressed in the ΔIVΔCEL mutant. The resulting strains were inoculated into *N. benthamiana* leaves and assayed for growth 6 days later. Growth of the ΔIVΔCEL mutant was substantially less than that of the ΔIV mutant, and as expected, the positive control clones *shcM-hopM1* and *shcE-avrE* significantly enhanced growth (Figure 3A). Growth was not enhanced by expression of *hopD1*, but it was by expression of *hopR1*. Thus, HopR1 has functional redundancy with CEL effectors.

The Δ*hopQ1-1*ΔCEL mutant is much more strongly reduced in growth in tomato than in *N. benthamiana* (Figure 1). One possible explanation for this is that HopR1 makes less of a contribution to bacterial growth in tomato than in *N. benthamiana*, thus reducing functional redundancy with CEL effectors. To test this hypothesis, we inoculated tomato leaves with the ΔIVΔCEL strain expressing *hopD1*, *hopR1*, *shcM-hopM1,* or *shcE-avrE*. As expected, HopD1 did not enhance growth, whereas HopM1 and AvrE did so strongly (Figure 3B). HopR1 produced a slight increase in growth that was not significantly different from the ΔIVΔCEL parent strain in the context of the higher variability in bacterial growth in tomato leaves. Although we cannot conclude that HopR1 makes absolutely no contribution to bacterial growth in tomato, it appears that the contribution of HopR1, relative to HopM1 and AvrE, is significantly less in tomato than in *N. benthamiana*.

### HopR1 is a member of an effector superfamily that is widely distributed in diverse phytopathogenic bacteria

**Figure 4:**
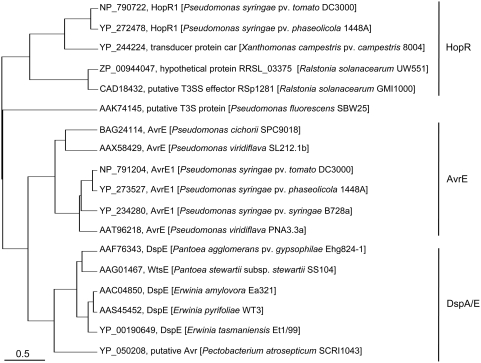
HopR1 is a member of an effector superfamily that is widespread among phytopathogens. A distance-based MUSCLE tree shows that HopR1 is in one of three major clades of the AvrE/DspA/E/HopR superfamily. The tree was generated from a structural alignment using the MUSCLE algorithm [33] and displayed using NJplot [73]. The lengths of the branches are measures of structural similarity.

BLAST-P with HopR1 from DC3000 generates hits to putative HopR1 homologs in *Xanthomonas campestris* pv. *campestris* and *Ralstonia solanacearum* (see http://www.ncbi.nlm.nih.gov/sutils/blink.cgi?pid=28868103). It also reveals a low quality hit (E-value = 0.042) to AvrE from *Pseudomonas cichorii*. We subsequently found that RSp1281, the HopR1 homolog from *R. solanacearum* GMI1000, generates BLAST-P hits with lower scores to AvrE from *P. viridiflava* and *P. syringae* (see http://www.ncbi.nlm.nih.gov/sutils/blink.cgi?pid=17549500). To investigate the relationship between AvrE and HopR1 in more detail, we performed a PSI BLAST analysis using the DC3000 HopR1 and stringent inclusion criteria (E-value<1.0E-10). Hits to AvrE occurred in the second iteration and hits to *E. amylovora* DspA/E occurred in the third iteration. A selection of third iteration PSI BLAST hits was made to maximize strain and species diversity and then used to generate an alignment and neighbor-joining tree using MUSCLE [33], which was subsequently displayed using NJplot (Figure 4). This analysis revealed that HopR1 is a member of a superfamily that is comprised of AvrE, DspA/E, and HopR families.

BLAST-P analysis also revealed a large number of hits at the C-terminus of the DC3000 HopR1 to metazoan cytoskeleton-interacting proteins, such as rootletin and myosin. This C-terminal domain only aligns for HopR1 homologs from DC3000, *P. syringae* pv. *phaseolicola* 1448a, and *X. campestris* pv. *campestris*. Also, analysis of the C-terminus of the *X. campestris* pv. *campestris* HopR1 homolog yielded a hit in CDD search to the PFAM02463 SMC_N terminal domain (cluster ID 410621). Alternatively, a more detailed analysis of the BLAST alignments suggests that the regions of similarity at the C-termini may be more generic, attributable to the presence of coiled-coil domains. Such domains are found in most of the BLAST-P hits to HopR as well as to SMC_N domains, and they have very regular primary structures. To check for the presence of coiled-coil domains, members of the AvrE/DspA/E/HopR superfamily were analyzed using the coiled-coil predictor COILS [34]. The three HopR homologs with the extended C-terminus had coiled-coil domains in that region, which were strongly predicted by COILS. Additionally, most members of the family that were analyzed had a predicted coiled-coil domain near the middle of the protein (in the vicinity of residue 1000).

### HopR1 suppresses callose formation by the ΔIVΔCEL mutant in *N. benthamiana*

**Figure 5:**
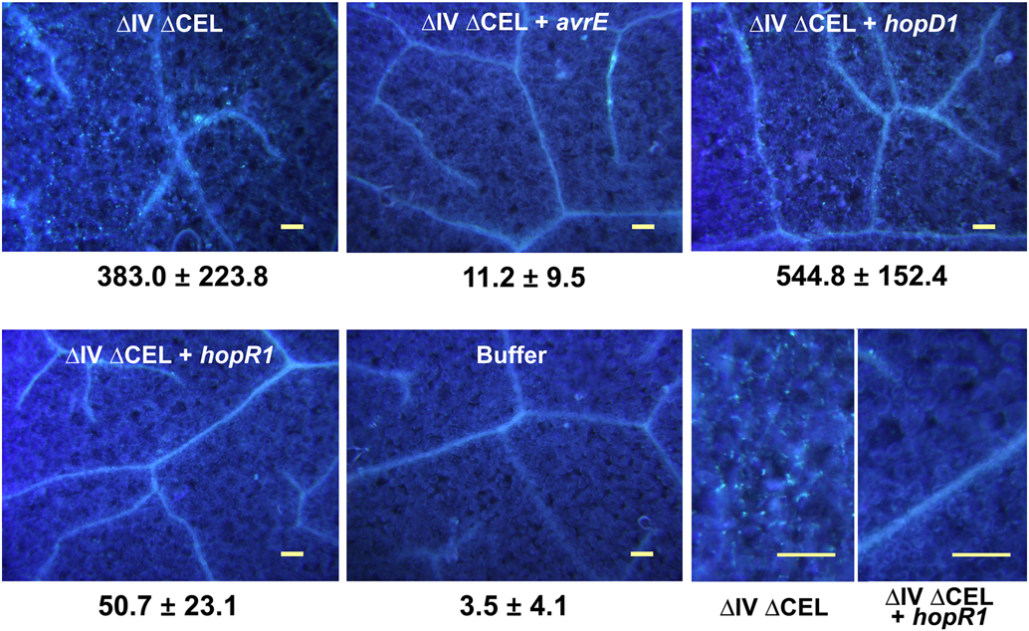
HopR1 suppresses callose formation by the ΔIVΔCEL mutant in *N. benthamiana*. *N. benthamiana* leaves were infiltrated with the ΔIVΔCEL mutant strains transformed with pBBR *P*_avrPto_ *avr/hop* expression constructs at 5×10^8^ CFU/ml with a blunt syringe. Aniline blue-stained callose depositions in *N. benthamiana* leaves were visualized by epifluorescence microscopy 12 h after inoculation. Numbers are the means and standard deviations of eight 1-cm^2^ microscopic fields of view. The panels in the lower right show enlargements of representative areas from leaf areas inoculated with the ΔIVΔCEL mutant and the mutant expressing *hopR1*. The experiment was repeated six times with similar results. The scale bar is 100 µm.

*P. syringae* T3SS-deficient mutants and DC3000 ΔCEL mutants are reduced in their ability to suppress the formation of callose deposits, which are associated with papillae that form as appositions inside plant cell walls and which provide a useful assay for cell wall-based PTI defenses [32],[35],[36]. Because the expression of *avrE in trans* has been shown to restore suppression of callose formation to a DC3000 ΔCEL mutant in Arabidopsis [32], we were curious to see if AvrE and HopR1 could function redundantly in the same assay in *N. benthamiana*. We accordingly inoculated *N. benthamiana* leaves with the ΔIVΔCEL mutant and with the mutant expressing *shcE-avrE*, *hopD1*, or *hopR1* under the direction of the *avrPto* promoter in the broad-host-range vector pBBR derivative pCPP5372 and after 12 h assayed for the formation of aniline blue-stained deposits by epifluorescence microscopy. The ΔIVΔCEL mutant elicited the formation of ca. 100-fold more callose deposits than was observed with the buffer mock inoculation (Figure 5). The ΔIVΔCEL mutant expressing *hopD1 in trans* elicited at least as many callose deposits, whereas the mutant expressing either *avrE* or *hopR1* expressed substantially fewer. The complementation assays presented in Figures 5 and 3, respectively, suggest that expression of *hopD1* in the ΔIVΔCEL mutant may elicit stronger defenses and interfere with growth in *N. benthamiana*, whereas expression of *hopR1* suppresses defenses and promotes growth in *N. benthamiana*.

### Deletion of the flagellin *fliC* gene rescues growth in *N. benthamiana* leaves of a Δ*avrPto*Δ*avrPtoB* mutant but not of a ΔIVΔCEL mutant

**Figure 6:**
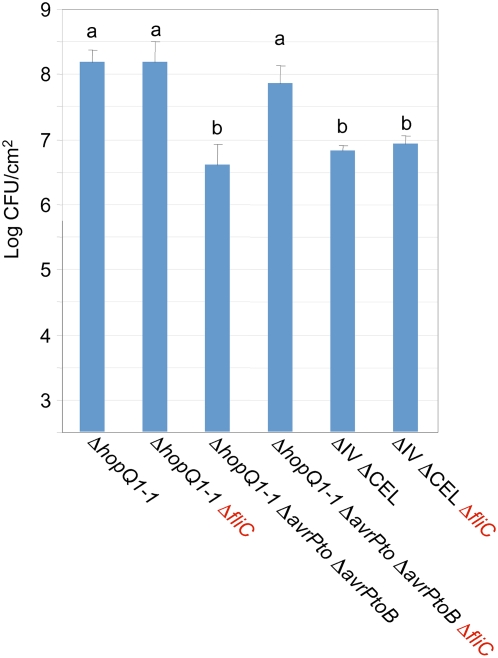
Deletion of the flagellin *fliC* gene rescues growth of Δ*avrPto*Δ*avrPtoB* but not ΔIVΔCEL mutants. *N. benthamiana* leaves were infiltrated with the indicated strains at 3×10^4^ CFU/ml (2.5 log CFU/cm^2^ leaf tissue) with a blunt syringe. Bacterial populations were determined from three 0.8-cm leaf discs 6 days post-inoculation. Results are the mean and standard deviation of bacterial populations collected from four leaf samples. Means marked with the same letter are not statistically different at the 5% confidence level based on Duncan's multiple range test. This experiment was repeated three times with similar results.

The work described above revealed that among the 18 effector genes occurring in clusters in the DC3000 genome, those in the CEL and *hopR1* in cluster IV make the largest contribution to the growth of Δ*hopQ1-1* DC3000 in *N. benthamiana*. Among the effector genes outside of the clusters, *avrPto* and *avrPtoB* are likely to be particularly important because an Δ*avrPto*Δ*avrPtoB* mutant had been shown to be significantly reduced in virulence in tomato [27]. To foster analysis of the Δ*avrPto*Δ*avrPtoB* mutant in *N. benthamiana* and comparison with effector gene cluster polymutants, we introduced the Δ*hopQ1-1* mutation into DC3000Δ*avrPto*Δ*avrPtoB* [27]. The Δ*hopQ1-1*, Δ*hopQ1-1*Δ*avrPto*Δ*avrPtoB*, and ΔIVΔCEL mutants were inoculated into *N. benthamiana* leaves and assayed for growth 6 days later. The Δ*hopQ1-1*Δ*avrPto*Δ*avrPtoB* and ΔIVΔCEL mutants were significantly reduced in growth relative to the Δ*hopQ1-1* strain (Figure 6). As previously shown with the Δ*avrPto*Δ*avrPtoB* mutant in tomato [27], growth of the Δ*hopQ1-1*Δ*avrPto*Δ*avrPtoB* was partially restored by complementation with *avrPtoB* (Figure S2). The Δ*hopQ1-1*Δ*avrPto*Δ*avrPtoB* and ΔIVΔCEL mutants were reduced in growth to a similar degree in *N. benthamiana* (Figure 6).

The primary function of the *P. syringae* effector repertoire is thought to be the suppression of PTI, and it appears that flagellin is a particularly important PAMP in *P. syringae*-plant interactions [37],[38]. To explore the relative role of the CEL-HopR1 and AvrPto/AvrPtoB effector groups in defeating flagellin-triggered immunity, we deleted the *fliC* flagellin gene from the three strains analyzed above in Figure 6. Loss of flagellin had no significant effect on the growth of the Δ*hopQ1-1* and ΔIVΔCEL strains, but it significantly enhanced the growth of the Δ*hopQ1-1*Δ*avrPto*Δ*avrPtoB* strain.

## Discussion

**Figure 7:**
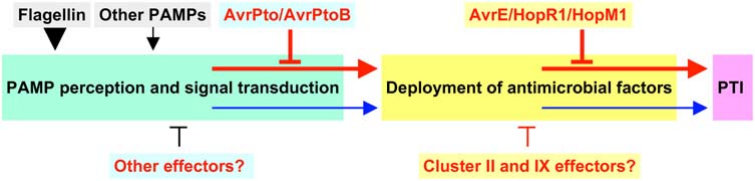
Proposed model for the role of redundant effector groups (REGs) in blocking distinct steps in PTI. The model depicts flagellin as the major DC3000 PAMP detected by *N. benthamiana*. A REG comprises effectors (e.g., AvrPto/AvrPtoB) that function redundantly to block a single high-level PTI process (e.g., PAMP perception). There is also intrinsic redundancy in each high-level PTI process (denoted by the red and blue arrows), which prevents the action of a single REG from completely blocking PTI. The existence of candidate minor members of REGs is revealed by observing further reductions in pathogen growth when minor member genes are deleted from mutants lacking the major members, as exemplified by clusters II and IX in the proposed AvrE/HopR1/HopM1 REG. The model predicts that (i) many effectors in the *P. syringae* pan-genomic effector super-repertoire can be assigned to REGs targeting a limited number of vulnerable high-level processes in PTI, (ii) any virulent strain will contain at least two REGs with at least one active member in each REG, and (iii) REGs can be used to functionally dissect vulnerable processes underlying PTI.

We have constructed combinatorial deletions involving all 18 of the *P. syringae* pv. *tomato* DC3000 T3SS effector genes that occur in genomic clusters and in two of the remaining 10 active effector genes. The successive removal of these genes has revealed that five effectors occurring in two REGs (redundant-effector groups) account for much of the ability of DC3000 Δ*hopQ1-1* derivatives to multiply in the model plant *N. benthamiana*. We will discuss general features of *P. syringae* effector repertoires, our combinatorial deletion strategy, the newly defined AvrE/DspA/E/HopR effector superfamily, and a model for pathogenesis based on each REG attacking a different high-level process in PTI (Figure 7).

### *P. syringae* effector repertoires

A consideration of the effector repertoires of other strains and the *P. syringae* effector pan-genome is useful in interpreting our results. *P. syringae* strains are host-specific and are assigned to more than 50 pathovars based largely on host specificity in the field. The effector repertoires have been established for fully sequenced strains in three pathovars – *P. syringae* pv. *tomato* DC3000 (bacterial speck of tomato, Arabidopsis and *N. benthamiana*), *P. syringae* pv. *phaseolicola* 1448A (halo blight of bean), and *P. syringae* pv. *syringae* B728a (brown spot of bean and *N. benthamiana*) – and recently for draft-sequenced *P. syringae* pv. *tomato* T1 (bacterial speck of tomato) [13],[14],[21],[39],[40]. In general, there are only a few effectors that appear to be universally deployed: AvrE, HopI1, AvrPtoB (or other members of the HopAB family), and HopAF1 (HopM1 and HopAA1 also appear universal but are truncated in many strains) [13].

Beyond these universal effectors, the repertoires are remarkably diverse, which is consistent with the observation that effector genes are typically associated with mobile genetic elements and horizontally acquired islands in the genome, and there is no evidence that the effector genes in any island (other than the CEL) are clustered by function [23]. Importantly, there appears to be no pattern among the variable effectors in the sequenced strains that would predict host range [13],[20],[39], and well-studied variable effectors such as AvrPto appear to have the same PTI-suppressive activity in diverse plants. Similarly, deleting DC3000 effector cluster IX (carrying 4 variable effector genes) results in reduced symptom formation in Arabidopsis, tomato, and *N. benthamiana* [20], which is consistent with the partially reduced virulence of *hopAO1* mutants that was previously observed [41]–[43]. Interestingly, the DC3000 *hopAO1* mutants showed a significant reduction in growth per se in tomato and Arabidopsis, which we did not observe with ΔIX mutants in *N. benthamiana* except in combination with other effector gene cluster deletions. It will be interesting to see in the future if this possible difference in the importance of HopAO1 results from differences in *N. benthamiana* targets of HopAO1 or functional redundancy with other effectors that work better in *N. benthamiana*. In this regard, it is noteworthy that *N. benthamiana* is unusually susceptible to a wide range of plant viruses and other pathogens, although importantly, not all *P. syringae* pathovars are virulent on *N. benthamiana* [20],[44].

It is also important to note here that deletion of individual effector genes from *P. syringae* pv. *syringae* B728a resulted in increased virulence indicative of weak avirulence activity, in a differential manner, in the two susceptible plants tested: bean and *N. benthamiana* [39]. This finding further supports the widespread importance of effector interactions with the R-protein surveillance system, which we consider to be fundamentally more polymorphic than the virulence targets of the effectors. However, there is clearly some polymorphism in the PTI defense system, as exemplified by the lack of a functional EFR (elongation factor-Tu receptor) in *N. benthamiana* [45]. Similarly, our observation that HopR1 has a stronger capacity to promote growth in *N. benthamiana* than in tomato would be consistent with polymorphism between the PTI defenses of these plants. Because the distribution of effectors in the sequenced strains shows no relationship with host range, we favor the hypothesis that most effectors are “generalists” with targets that may sometimes be polymorphic, rather than “specialists” with targets unique to different plant groups and therefore essential for the inclusion of that plant group in the pathogen's host range. In summary, these observations suggest that any effector in the super-repertoire of the *P. syringae* pan-genome (which currently comprises 42 effector families: http://pseudomonas-syringae.org) could promote virulence in many (if not all) plants, barring detection by an R protein.

If the many effectors in the pan-genome are functionally interchangeable in a wide range of potential hosts, what selection pressures, if any, constrain the composition of effector repertoires other than evasion of R-protein surveillance? We suggest that two types of relationships among effectors might underlie the canonical observation that effectors are collectively essential but individually dispensable. First, the majority of effectors may act independently to defeat PTI through a massively redundant “death by a thousand cuts” strategy. Second, much of the redundancy may be organized around REGs, which attack host defenses in some coordinated way. The key to differentiating between these models is to determine the extent of interplay among effectors.

### Approaches to identifying interplaying effectors

Effector interplay can be detected through both gain-of-function and loss-of-function experiments. The former have been particularly useful in identifying potential interplay involving defined pathogenic processes, such as ETI suppression. For example, nonpathogenic *P. fluorescens* expressing a cloned *P. syringae* T3SS and HopA1 (an effector with avirulence activity in *N. tabacum*) was used in a survey for DC3000 effectors that could suppress the HopA1-triggered hypersensitive response when expressed *in trans* [46]. A recent, powerful example of the gain-of-function approach involves the six *Salmonella* effectors that manipulate the host cytoskeleton to promote pathogen internalization into non-phagocytic intestinal cells [47]. Augmented expression of effector pairs in interacting bacteria and transfected cultured cells revealed both synergistic and antagonistic relationships among these effectors in promoting internalization.

Faced with 28 effectors and the resulting vast number of potential gain-of-function combinations, we chose to identify interplaying effectors by constructing combinatorial deletions and assaying for reduced bacterial growth in planta. By exploiting the clustering of effector genes we could eliminate 18 effectors with just six genetic manipulations, and the anticipated internal redundancy within the CEL promised to further simplify the problem. Importantly, any virulence phenotypes resulting from deletions could be cleanly attributed to the natural function of the relevant effectors (expressed natively and in the context of the remaining repertoire and other virulence factors), which contrasts with experiments involving heterologous expression. By assaying for bacterial growth, we assessed the overall parasitic ability of mutants independently of any disease sub-processes, such as lesion formation, chlorosis, or defense suppression, which can be uncoupled from each other and from growth in complex ways. *N. benthamiana* is ideal for growth assays because of its large and easily inoculated leaves, the reproducibility of the data, and the possibility of rapidly testing the role of candidate host factors using virus-induced gene silencing. Importantly, our approach of constructing multi-effector deletions, beginning with our previously reported CEL deletion, produces DC3000 derivatives with easily assayed phenotypes for functional analysis of individual effectors through complementation in near-native backgrounds [30],[32].

### The AvrE/DspA/E/HopR superfamily

Recognition of the AvrE/DspA/E/HopR superfamily was one useful outcome of our work. AvrE was the first effector to be characterized in DC3000 [48], but the AvrE/DspA/E/HopR superfamily and the relationship between HopR1 and AvrE in DC3000 had not been fully recognized before. HopR1 (formerly HolPtoR) had previously been observed to be a homolog of the *R. solanacearum* GMI1000 RSp1281 and *X. campestris*. pv. *campestris* ATCC33913 Xcc1089 proteins [3],[49], and RSp1281 orthologs recently were noted in several sequenced plant pathogens [50]. The PSI-BLAST and MUSCLE analyses performed here define the AvrE/DspA/E/HopR superfamily and suggest that it is present in virtually all groups of T3SS-dependent plant pathogens with the notable exception of some xanthomonads that carry the AvrBs3 effector family, which is a highly amplified and important effector family that behaves like a REG in these strains [51]. Proteins in the AvrE/DspA/E/HopR superfamily are large (ca. 2,000 amino acids), and regions of similarity are found across the length of the proteins, although the predicted C-terminal coiled-coil domain in the DC3000 HopR1 and Xcc1089 proteins is lacking from RSp1281 and AvrE.

There is evidence for at least one member of each family in the AvrE/DspA/E/HopR superfamily contributing to virulence. For example, in the HopR family, the *X. campestris* pv. *campestris* 8004 ortholog was identified in a transposon screen for mutants with reduced virulence [52]. DspA/E has been shown to be essential for the virulence of *Erwinia amylovora* [53], and it is partially interchangeable with the DC3000 AvrE [54]. AvrE was the one effector identified in a transposon screen for DC3000 mutants with reduced virulence in a sensitive Arabidopsis dip inoculation assay [55]. It is also noteworthy that the RSp1281, *hopR1*, and *avrE* genes are considered to be ancient constituents of their respective genomes, which further points to the importance of this superfamily in plant pathogenesis [49],[50].

### The REG-based architecture of the DC3000 effector repertoire

The pattern of growth defects attending our combinatorial deletions does not support the hypothesis that the DC3000 effector repertoire defeats *N. benthamiana* through a “death by a thousand cuts” strategy. Notably, deletions involving a majority of the clustered effectors had no impact on growth. Instead, the deletion phenotypes revealed a functional architecture in the effector repertoire that is structured around REGs, whose members are self-identified by phenotype. For example, HopR1 makes a significant contribution to growth only in the context of the CEL deletion, and was thus determined to be in the same REG (and only later did we recognize the sequence similarity between AvrE and HopR1). The further finding that deletions involving AvrE/HopR1/HopM1 and AvrPto/AvrPtoB produced equivalently strong reductions in bacterial growth in *N. benthamiana* suggests a model for DC3000 pathogenesis involving these two REGs targeting different high-level processes in PTI (Figure 7).

According to this model, AvrPto and AvrPtoB functionally overlap in blocking PAMP perception and signal transduction. Therefore, removing both of these effectors is necessary to fully unblock PAMP signal processing for robust PTI development in infected plants. This part of the model is supported by (i) recent biochemical evidence that AvrPto and AvrPtoB target the FLS2/BAK1 complex needed for flagellin perception [28],[29], (ii) our observation that deleting *fliC* compensates for the loss of these two effectors, and (iii) the observation that flagellin may be a particularly important PAMP in bacterial interactions with *N. benthamiana* because this plant lacks the EFR pattern recognition receptor kinase needed for recognition of another important bacterial PAMP, elongation factor Tu [45].

AvrE, HopR1, and HopM1 are proposed to overlap in blocking a different high-level process in PTI: deployment of antimicrobial factors, including cell wall defenses associated with callose formation. This hypothesis is supported by (i) the sequence similarity of AvrE and HopR1, (ii) multiple observations of functional redundancy between AvrE and HopM1 [26],[32] (and now HopR1), (iii) the evidence that HopM1 blocks the vesicle trafficking that is likely important for deploying antimicrobial factors [56], (iv) multiple observations that proteins in the DspA/E, AvrE, and HopM1 families can promote cell death in compatible host cells [26],[31],[57],[58], which is consistent with their disruption of a common cellular process, such as vesicle trafficking, and contrasts with the lack of such an effect by AvrPto and AvrPtoB in compatible hosts, (v) the failure of the *fliC* deletion to compensate for the loss of the AvrE/HopR1/HopM1 REG, and (vi) the ability of HopR1 to function similarly to AvrE in suppressing callose formation. Also, it is noteworthy that a knockout of the AtMIN7 target of HopM1 only partially restores growth of ΔCEL DC3000 in Arabidopsis, whereas treatment of Arabidopsis with Brefeldin A, an inhibitor of vesicle trafficking, restores growth to wild-type levels [56]. Although this effect was attributed to additional activities of HopM1, we find attractive the alternative hypothesis that the inhibition of vesicle trafficking by the CEL results from AvrE as well as HopM1.

What selection pressures drive the formation of a REG and expansion of its membership? Recent advances support the following scenario for the evolution of the AvrPto/AvrPtoB REG [29],[59]: plants countered the PTI-suppressive kinase-inhibitory activity of these effectors by evolving kinase decoys guarded by R proteins, and the resulting ETI drove pathogen evolution for the acquisition and substitution of alternative effectors in the same REG.

The coevolutionary dynamics of the AvrE/HopR1/HopM1 REG are less clear. These are all considered ancient effectors in *P. syringae* [49], but intriguingly, no cognate R protein or case of gene-for-gene, race-specific resistance against these effectors has been reported. For example, *avrE* was originally cloned from *P. syringae* pv. *tomato* PT23 on the basis of its ability to confer avirulence to *P. syringae* pv. *glycinea* race 4 in soybean [60], and it was later found to confer such avirulence to all cultivars tested [61]. It is possible that ancient effectors like AvrE are indeed under R-protein surveillance, but this is obscured by ETI suppressors. However, our combinatorial deletion analysis has not supported this hypothesis. In contrast, the observation that AvrE, DspA/E, WtsE, and HopM1 can promote cell death in compatible hosts raises the possibility that the ETI system may detect general perturbations in the process (most likely vesicle trafficking) that is targeted by this REG in a manner that is independent of the specific structure and mechanistic details of the individual members of the group.

While the existence of multiple effectors within each REG would confer robustness to pathogens that attack a limited set of vulnerable processes in plants, our data suggest a high-level redundancy among these targets that provides robustness to the defense system. That is, it appears that no single REG is sufficient to confer maximal pathogen growth. As depicted in Figure 7, PAMP perception and antimicrobial deployment are serial processes, and each process has a secondary “backup” pathway, which may be targeted by other effectors. Thus, the strong reduction in bacterial growth observed with the Δ*avrPto*Δ*avrPtoB* mutant indicates that these effectors indeed target the major process in PAMP perception in *N. benthamiana*. But there apparently exists a backup pathway for PAMP perception. Otherwise the ΔIVΔCEL mutant and other effector gene cluster mutants that we constructed would all grow to wild-type levels because AvrPto/AvrPtoB would always block the first step in the PTI process. That is, AvrPto/AvrPtoB are necessary for robust growth in *N. benthamiana* (as seen with the Δ*avrPto*Δ*avrPtoB* mutant), but they are not sufficient for growth (as seen with the ΔIΔIIΔIVΔCELΔIXΔX mutant).

The converse argument can be made for redundancy in the high-level process of deploying antimicrobial factors, which is strongly but incompletely blocked by the AvrE/HopR1/HopM1 REG. We postulate that the apparent redundancy in high-level PTI processes requires *P. syringae* to deploy multiple REGs to adequately suppress PTI. It is also worth noting here that REGs may differ in their internal redundancy and relative importance in different plants. Thus, the AvrE/HopR1/HopM1 REG appears to have less internal redundancy in tomato than in *N. benthamiana* because of the limited efficacy of HopR1 in tomato, and in contrast AvrPto/AvrPtoB appear to be more important in *N. benthamiana* than in tomato (Figures 1 and 6) [27]. Quantitative differences in the defense systems of potential hosts may be another factor promoting the expansion and diversity of *P. syringae* effector repertoires.

It is possible that combinatorial deletions involving the eight remaining effector genes outside of the clusters will reveal additional REGs in DC3000. The widespread effectors HopE1, HopI1, and HopAF1 are of particular interest. HopAI1, although apparently not actively deployed by DC3000, is a phosphothreonine lyase that inactivates PTI-associated mitogen-activated protein kinases (MAPKs) and therefore represents a good candidate for another *P. syringae* effector targeting PAMP perception and signaling [62],[63]. It is also possible that the minimal set of DC3000 effectors needed to defeat plants will include REG representatives plus other effectors that act more globally to remodel plant metabolism, such as HopU1 and HopT1-1, which ADP-ribosylate RNA-binding proteins and suppress the microRNA pathway, respectively [18],[64]. Additional complexities include the apparent multi-domain structure of many effectors and potential membership in more than one REG. 

A comprehensive picture of the molecular activities of effectors inside plant cells is beginning to develop [1],[65]. Our combinatorial deletion analysis complements these studies and provides resources and testable hypotheses for further analysis of effector repertoires. For example, DC3000 REG polymutants could be used to screen effectors in the *P. syringae* pan-genome or in fungi and oomycetes (appropriately modified for delivery by the T3SS [6],[66]) for their high-level PTI targets, as indicated by REG-specific rescue of bacterial growth. Indeed, there is partial precedence for this in the ability of the *Hyaloperonospora parasitica* effector ATR13 to restore bacterial growth and callose suppression in Arabidopsis to a DC3000 ΔCEL mutant [66]. Although ATR13 also enhances the virulence of wild-type DC3000 in Arabidopsis, it is possible that an analysis of the relative ability of this effector to restore growth in planta to the Δ*avrPto*Δ*avrPtoB* and ΔIVΔCEL mutants could reveal a clear difference indicative of the primary high-level target. Hopefully, approaches such as this, which exploit DC3000 and its well studied effector repertoire, will accelerate elucidation of the effector repertoires produced by diverse pathogens and eventually guide the deployment of more durable *R* genes for protecting crops against multiple pathogens.

## Materials and Methods

### Bacterial strains

2*E. coli* and DC3000 strains and plasmids used in this study are listed in Table 1. Plasmid maintenance and manipulations were typically conducted with either *E. coli* DH5α or TOP10. Invitrogen Gateway maintenance and manipulations were conducted in *E. coli* DB3.1. *E. coli* S17-1 and SM10 were used for conjugations.

**Table 1:** Bacterial strains and plasmids used in this study

Designation	Genotype	Relevant Features	Source Reference
***E. coli***			
DH5α	F^−^ Φ80*lacZ*ΔM15 Δ(*lacZYA*-*argF*)U169 *recA1*; *endA1 hsdR17*(r_k_^−^ m_k_^+^) *phoA supE44 thi-1 gyrA96 relA1* λ^−^	Nx^R^	Invitrogen
TOP10	F*^−^ mcrA* Δ(*mrr-hsdRMS-mcrBC*) Φ80*lacZ*ΔM15 Δ*lacX74 recA1 araD139* Δ(*ara-leu*)7697 *galU galK rpsL endA1 nupG*	Sm^R^	Invitrogen
DB3.1	F^−^ *gyrA462 endA1*Δ(*sr1*-*recA*) *mcrB mrr hsdS20* (r_B_-, m_B_-) *supE44 ara-14 galK2 lacY1 proA2 rpsL20 xyl-5* λ^−^ *leu mtl1*	Sm^R^, *ccdB* ^R^	Invitrogen
S17-1	*thi pro hsdr^−^ hsdM^+^ recA* (chr::RP4 2-Tc::Mu- Km::Tn*7*)	RP4, Tp^R^, Sp^R^	[74]
SM10	*thi thr leu suIII* (chr::RP4 2-Tc::Mu)	RP4, Km^R^	[74]
***P. syringae*** ** pv. ** ***tomato*** ** DC3000**	Wild type	Rf^R^, Ap^R^	
CUCPB5113	Δ*hrcQ_B_-hrcU*::ΩSp^R^	T3SS^−^, Sp^R^	[26]
CUCPB5460	Δ*hopQ1-1*	ΔQ	[20]
CUCPB5501	Δ*hopQ1-1* Δ*avrE-shcN*	ΔQΔCEL	(This study)
CUCPB5452	Δ*hopC1-hopH1*::FRT Δ*hopD1-hopR1*::FRT; Δ*hopAA1-2-hopG1*::FRT pDC3000A^−^B^−^	ΔIIΔIVΔIXΔX	[20]
CUCPB5459	Δ*hopU1-hopF2* Δ*hopC1-hopH1*::FRT Δ*hopD1-hopR1*::FRT Δ*hopAA1-2-hopG1*::FRT pDC3000A^−^B^−^	ΔIΔIIΔIVΔIXΔX	(This study)
CUCPB5500	Δ*hopU1-hopF2* Δ*hopC1-hopH1*::FRT Δ*hopD1-hopR1*::FRT Δ*avrE-shcN*; Δ*hopAA1-2-hopG1*::FRT pDC3000A^−^B^−^	ΔIΔIIΔIVΔCELΔIXΔX	(This study)
CUCPB5515	Δ*hopD1-hopR1*::FRT Δ*avrE-shcN*	ΔIVΔCEL	(This study)
CUCPB5516	Δ*hopD1-hopR1*::FRT Δ*avrE-shcN* pDC3000A-B-	ΔIVΔCELΔX	(This study)
CUCPB5517	Δ*hopC1-hopH1*::FRT Δ*hopD1-hopR1*::FRT Δ*avrE-shcN* pDC3000A-B-	ΔIIΔIVΔCELΔX	(This study)
CUCPB5518	Δ*hopC1-hopH1*::FRT Δ*hopD1-hopR1*::FRT Δ*avrE-shcN* Δ*hopAA1-2-hopG1*::FRT; pDC3000A^−^B^−^	ΔIIΔIVΔCELΔIXΔX	(This study)
CUCPB5529	Δ*hopQ1-1* Δ*hopAA1-2-hopG1*::FRT	ΔQΔIX	(This study)
CUCPB5530	Δ*hopQ1-1* Δ*avrE-shcN*; Δ*hopAA1-2-hopG1*::FRT	ΔQΔCELΔIX	(This study)
CUCPB5440	Δ*hopD1-hopR1*::FRT	ΔIV	[20]
CUCPB5447	Δ*hopD1-hopR1*::FRT Δ*hopAA1-2-hopG1*::FRT	ΔIVΔIX	[20]
CUCPB5531	Δ*hopD1-hopR1*::FRT Δ*avrE-shcN*; Δ*hopAA1-2-hopG1*::FRT	ΔIVΔCELΔIX	(This study)
CUCPB5538	Δ*hopD1-hopR1*::FRT Δ*avrE-shcN*; Δ*hopAA1-2-hopG1*::FRTSp^R^ pDC3000A^−^B^−^	ΔIVΔCELΔIXΔX, Sp^R^	(This study)
CUCPB5539	Δ*hopC1-hopH1*::FRT Δ*hopD1-hopR1*::FRT Δ*avrE-shcN* Δ*hopAA1-2-hopG1*::FRT	ΔIIΔIVΔCELΔIX	(This study)
CUCPB5484	Δ*hopQ1-1* Δ*fliC*::FRT	ΔQΔ*fliC*	(This study)
CUCPB5489	Δ*hopQ1-1* Δ*avrPtoB*::*nptII* Δ*avrPto*::ΩSp^R^	ΔQΔ*avrPto*; Δ*avrPtoB*, Km^R^, Sp^R^	(This study)
CUCPB5514	Δ*hopQ1-1* Δ*avrPtoB*::*nptII* Δ*avrPto*::ΩSp^R^ Δ*fliC*::FRT	ΔQΔ*avrPto*; Δ*avrPtoB*Δ*fliC*, Km^R^, Sp^R^	(This study)
CUCPB5541	Δ*hopD1-hopR1*::FRT Δ*avrE-shcN* Δ*fliC*::FRTGm^R^	ΔIVΔCELΔ*fliC*, Gm^R^	(This study)
**Plasmids**			
pK18*mobsacB*	pMB1 *mob sacB*	Suc^S^, Km^R^	[75]
pCPP5610	pK18*mobsacB*::Δ*hopU1-hopF2*	Suc^S^, Km^R^	(This study)
pCPP5734	pK18*mobsacB*:: Δ*avrE-shcN*	Suc^S^, Km^R^	(This study)
pCPP5917	pK18*mobsacB*::Δ*hopAA1-2-hopG1*::FRTSp^R^	Suc^S^, Km^R^, Sp^R^	(This study)
pCPP5729	pK18*mobsacB*Gm^R^::Δ*hopQ1-1*	Suc^S^, Km^R^	(This study)
pCPP5615	pK18*mobsacB*::Δ*fliC*::FRTGm^R^	Suc^S^, Km^R^, Gm^R^	(This study)
pCPP5264	pRK415 *C1 FLP*	Tc^R^,	[11]
pCPP5372	pBBR1MCS5 *P*_avrPto_- Gateway RFB-HA	Gm^R^, Cm^R^	[12]
pCPP5588	pENTRSD/D-TOPO::*hopD1*	Km^R^	[71]
pCPP5951	pENTRSD/D-TOPO::*hopR1*	Km^R^	(This study)
pCPP5233	pENTRSD/D-TOPO::*shcM hopM1*	Km^R^	(This study)
pCPP5912	pENTRSD/D-TOPO::*shcE avrE*	Km^R^	(This study)
pCPP5616	pENTRSD/D-TOPO::*avrPtoB*	Km^R^	[71]
pCPP5702	pUCP26::ΩKm *P_avrpto_ avrPto-cya*	Gm^R^ Km^R^	[11]

### Media and culture conditions

DC3000 strains were routinely grown in King's B (KB) medium at 25 or 30°C [67]. *E. coli* strains were routinely grown on LB media at 37°C. Growth of DC3000 and derivatives was also compared on two defined media: mannitol-glutamate medium (MG) and Hrp-minimal medium (HMM) [68],[69]. Antibiotics and additives were used at the following final concentrations (µg/ml) unless otherwise noted: ampicillin (Ap), 100; kanamycin (Km), 50; gentamicin (Gm), 5; spectinomycin (Sp), 50; rifampicin (Rf), 50; tetracycline (Tc), 20; 5-bromo-4-chloro-3-indolyl-beta-D-galactopyranoside (X-gal), 40.

### Recombinant DNA techniques

DNA manipulations and PCR were conducted according to standard protocols [70]. Plasmid DNA was purified using the QIAprep Spin Miniprep Kit from Qiagen (Valencia, CA). Genomic DNA was prepped using the Wizard Genomic DNA Purification Kit from Promega (Madison, WI). DNA gel extraction and DNA enzyme reaction cleanups were conducted using the Gel DNA Recovery Kit and Clean-up and Concentrator Kit from Zymo Research (Orange, CA). DNA restriction and modification enzymes were from New England Biolabs (Ipswich, MA) and were used according to the manufacturer's recommendations. Invitrogen (Carlsbad, CA) Gateway recombination was conducted with LR clonase I or LR clonase II from Invitrogen as recommended by the manufacturer. PCR was routinely conducted using Takara Ex Taq Polymerase Premix from Takara Mirus Bio (Otsu, Shiga, Japan). PCR primers were obtained from IDT (http://www.idtdna.com). Primers and primer sequences are listed in Table 2. DNA sequencing was conducted at the Cornell University Biotechnology Resource Center using an Applied BioSystems 3730xl DNA Analyzer. Sequences were analyzed using the Vector NTI software package from Invitrogen.

**Table 2:** Oligonucleotide primers used in this study

Primers	5′ → 3′ Sequences	Restriction Enzyme Sites
P2308	CACCGGATCCCACTGCGGTACCGAC	*Bam*HI
P2309	ACCTCTAGATTTGAGTCCATGAAGG	*Xba*I
P2310	GCGTCTAGAGGTGCCGCAAATATTACC	*Xba*I
P2311	CGCAAGCTTCGAAAAAGTGTTGGG	*Hin*dIII
P2312	GCACCATGTGCCTGACCG	
P2313	TCGATGGCCTGCCACTGC	
P2370	CACCTCTAGATATTCAGATGAGCTTCATTGG	*Xba*I
P2371	GCCGACTAGTTTTAGGGTTTGCACTAATATC	*Spe*I
P2372	ATTAACTAGTGGTGAATCTCTCTGTCCATTT	*Spe*I
P2373	TAATTCTAGAGTCTACGGCATGGTTAAGCT	*Xba*I
P2501	CAGCGCCACCTACGATGAGT	
P1967	GAGGCTGCAGACAACGAACTGAACG	
P1968	GAACGGCTCGATCAGGCCCA	
P2299	GGCCACGTCGGGATATTG	
P2300	ATCGGAGGGATCGACAGC	
P2245	CACCCGTTGAAGCGGCCAACAC	
P2261	TAATCCTAGGAGCCATGATGAATTCCTC	*Avr*II
P2262	TAATCCCGGGCAGTAATATCGGCATGAGTT	*Xma*I
P2248	GAGGTCTTGTCGATCGCGACACTGT	
P2253	ATTACCTAGGGTGTAGGCTGGAGCTGCTTC	*Avr*II
P2326	ACCTTCCTGCCGCGCAAAGA	
P2327	CGAGTTGATCTTGTCGCGCACT	
P2502	CACC ATGGTCAAGGTTACCTCTTC	
P2505	CACGTTATCGAGTTCGCCCCA	
P1932	CACCATGACCAACAATGACCAGTACCA	
P1931	ACGCAAGTCAAGCAAGCC	
P2506	CACCATGACGATGAAAACTTCGCAAC	
P2507	TCAAGCCTGTAAAAAAGCACGCGCTT	
P2508	TAATGGCGCGCCAGCTCTTCAGTTCGAACCCCTCTT	*Asc*I
P2509	TAATGGCGCGCCAGAGAGATTCACCGTGCAGTCACCA	*Asc*I

### Plasmid and strain construction

DC3000 strain deletions were made with pK18*mobsacB* constructs essentially as described previously [11]. DC3000 strains were conjugated on sterile nitrocellulose squares on LM medium with *E. coli* S17-1 or *E. coli* SM10 carrying pK18*mobsacB* deletion constructs. DC3000 merodiploid transconjugates were selected with KB Rf Ap Km augmented with additional antibiotics when needed. Merodiploids were spread on KB Rf with 10% sucrose to counter-select the integration. For marked deletions, sucrose counter-selection plates were augmented with the appropriate antibiotics to select for the mutant population. Suc^R^ colonies were screened by PCR.

pCPP5610 was created by PCR amplification of 1.5 and 1.0-kb flanks to *hopU1* and *hopF2* with P2308/P2309 and P2310/P2311 respectively. The PCR fragments were digested with XbaI and ligated with T4 ligase. The 2.5 kb ligation product was gel purified, digested with BamHI and HinDIII and cloned into BamHI and HindIII digested pK18*mobsacB.* pCPP5610 was used to delete cluster I from CUCPB5452 to create CUCPB5459. The deletion was confirmed by PCR with P2312/P2313.

pCPP5734 was created by PCR amplification of 0.9 and 1.1-kb flanks to *shcN* and *avrE* with P2370/P2371 and P2372/P2373 respectively. The PCR fragments were digested with EcoRI and ligated with T4 ligase. The 2.0-kb ligation product was gel purified, digested with XbaI and cloned into XbaI digested pK18*mobsacB.* pCPP5734 was used to delete cluster VI/CEL from CUCPB5460, CUCPB5459, CUCPB5440, CUCPB5442, CUCPB5450, CUCPB5452 and CUCPB5451 to create CUCPB5501, CUCPB5500, CUCPB5515, CUCPB5516, CUCPB5517, CUCPB5518 and CUCPB5539 respectively. The deletions were confirmed by PCR with P2373/P2501.

pCPP5917 was created by sub-cloning the cluster IX deletion construct from pCPP5397 [11] with EcoRI into EcoRI digested pK18*mobsacB.* pCPP5917 was transformed into *E. coli* SM10 and used to delete cluster IX from CUCPB5460, CUCPB5501, CUCPB5515 and CUCPB5516 to create CUCPB5529, CUCPB5530, CUCPB5531, CUCPB5538 respectively. Merodiploid transconjugants were selected with KB Rf Ap Km Sp. The FRTSp^R^ cassette was removed from CUCPB5529, CUCPB5530 and CUCPB5531 by transformation and curing of the unstable FLP expression vector pCPP5264 [11]. The deletions were confirmed by PCR with P1967/P1968.

pCPP5729 was created by PCR amplification of the FRTGm^R^ cassette with P2259/P2260 from pCPP5209 [11]. The cassette was digested with XbaI and sub-cloned into pCPP5608 [20] so it could be used to make mutants in Km^R^ strains by screening for Gm^R^ and Gm^S^. Cloning with XbaI destroys the FRT sites. pCPP5729 was used to delete *hopQ1-1* from DC3000 Δ*avrPto*Δ*avrPtoB* and CUCPB5467 to make CUCPB5484 and CUCPB5489 respectively. The deletions were confirmed with by PCR with P2299/P2300.

pCPP5615 was created by PCR amplification of 1.1 and 1.0-kb flanks to *fliC* with P2245/P2261 and P2248/P2262 respectively and PCR amplification of the FRTGm^R^ cassette from pCPP5209 [11] with P2253/P2260. The three PCR products were digested with AvrII and XmaI and ligated with T4 ligase. The 3.4-kb product was gel purified and TOPO cloned into pCR2.1-TOPO. The deletion construct was then sub-cloned by SalI and SphI digestion into SalI/SphI digested pK18*mobsacB.* pCPP5615 was used to delete *fliC* from DC3000, CUCPB5489 and CUCPB5515 to create CUCPB5467, CUCPB5514 and CUCPB5541 respectively. The FRTGm^R^ cassette was removed from CUCPB5467and CUCPB5514 by transformation and curing of the unstable FLP expression vector pCPP5264 [11]. The deletions were confirmed by PCR with P2326/P2327 and loss of motility on a KB swim plate with 0.2% agar.

*hopR1* and *shcM hopM1* were PCR amplified with P2502/P2505 and P1932/P1931 respectively and TOPO cloned into pENTR/SD/D-TOPO (Invitrogen) to create pCPP5951 and pCPP5233 respectively. *shcE* with its stop codon was PCR amplified with P2506/P2507 and TOPO cloned into pENTR/D/SD-TOPO. *avrE* with its native SD but without its stop codon was PCR amplified with P2508/P2509 digested with AscI and cloned in-frame into the AscI digested pENTR/D/SD-*shcE* to create pCPP5912. All effector and chaperone clones were confirmed by DNA sequencing. pBBR *P*_avrPto_ constructs for complementation were made by LR recombination between pCPP5372 [12] and entry vectors carrying *hopR1, shcM-hopM1*, *shcE-avrE*, *hopD1*, or *avrPtoB* [71]. Plasmids were introduced into DC3000 derivatives by electroporation [72].

### Virulence assays

Virulence assays were conducted essentially as described previously [20]. *N. benthamiana* and tomato (*Solanum lycopersicum* cv. Moneymaker) were grown under greenhouse conditions until 4–5 weeks post-germination. Primary streaks of DC3000 strains were made from isolated colonies onto KB plates and grown overnight at room temperature. The plates were then spread with 100 µl sterile KB and incubated overnight at room temperature to produce even bacterial lawns. Cells were scraped from plates with a sterile loop and suspended in 10 mM MgCl_2_ 100 mM sucrose to a final OD_600_ of 0.3 (3×10^8^ CFU/ml). Bacterial suspensions were diluted to 3×10^4^ CFU/ml in 10 mM MgCl_2_ for syringe infiltration or 3×10^5^ CFU/ml in dH_2_O and 0.02% Silwet for dip inoculation. The bacterial concentrations of the suspensions were verified by plate count. Tomato and *N. benthamiana* syringe infiltrations were conducted by infiltrating expanded leaves using a dissecting needle and a blunt syringe. *N. benthamiana* dip inoculations were conducted by inverting whole plants into bacterial suspensions and gently agitating for 30 sec. The inoculated plants were incubated with a 12 h light cycle at 20–25°C with medium humidity for tomato and high humidity for *N. benthamiana*. Bacterial populations were assessed at three days post-inoculation for tomato and six days post-inoculation for *N. benthamiana.* Three 0.8-cm leaf discs were harvested with a cork borer from each infiltration area. The discs were ground with a sterile mortar and pestle into 0.3 ml 10 mM MgCl_2_ 100 mM sucrose, diluted and plated to determine CFU cm^−2^. The data for each figure presented are obtained from a single experiment with a single inoculum preparation, and each experiment was repeated as indicated.

### Callose staining

Six-week post-germination *N. benthamiana* leaves were infiltrated with a bacterial suspension with an OD_600_ of 0.5. Leaf disks were collected 12 hr after infiltration, cleared with 95% ethanol, and stained for callose with 0.1% aniline blue in 150 mM K_2_HPO_4_ (pH 9.5) as previously described [36]. The stained leaf disks were observed by epifluorescence microscopy under ultraviolet light. The numbers of callose deposits per 1 cm^2^ microscopic field were counted in randomly coded samples from eight leaf disks using images captured with an Olympus digital camera and its software (DP2-BSW). The callose deposit counts were then matched with the corresponding treatment codes for determination of the mean and standard deviation.

### Similarity tree construction

DC3000 HopR1 was used in a PSI BLAST with the inclusion parameter set to 1.0E^−10^. By the second iteration members of the AvrE clade and by the third iteration members of the DspA/E clade were identified. A selection of third iteration PSI BLAST hits were made to maximize strain and species diversity and were exported to the MUSCLE web server [33]. An alignment was generated with MUSCLE using default parameters (Figure S3), and a second iteration neighbor-joining tree was generated. The tree was displayed using NJplot [73].

### Accession numbers

*hopK1* (AAO53599), *hopY1* (AF458403), *hopU1* (AAO54045), *shcF* (AY321312), *hopF2* (AAO54046), *hopH1* (AAO54130), *hopC1* (AAO54131), *hopD1* (AAO54410), *hopQ1-1* (AF458396) , *hopR1* (AF458397), *hopAM1-1* (AAO54553), *shcN* (AE016853), *hopN1* (AAO54892), *hopAA1-1* (AAO54894), *shcM* (AE016853), *hopM1* (AAO54897), *shcE* (AE016853), *avrE* (AF232004), *hopB1* (AF232004), *hopAF1* (AAO55088), *avrPtoB* (AY074795), *avrPto* (AAO57459), *hopE1* (AY208297), *hopAA1-2* (AAO58156), *shcV* (AE016853), *hopV1* (AAO58158), *hopAO1* (AAO58160), *hopG1* (AY208296), *hopI1* (AAO58206), *hopA1* (AAO58779), *hopAM1-2* (AAO59032), *hopX1* (AAO59038),*shcO1* (NC_004633), *hopO1-1* (AF458392), *hopT1-1* (AF458399), RSp1281 (AL646053), Xcc1089 (NC_003902).

## Supporting Information

Figure S1 A CUCPB5500 derivative grows on minimal media and translocates AvrPto-Cya as well as wild type DC3000. (A) DC3000 and CUCPB5506 (a CUCPB5500 derivative with the phytotoxin coronatine biosynthesis *cfa* cluster deleted) were simultaneously streaked on mannitol glutamate (MG) and Hrp minimal medium (HMM) and then photographed 4 days later to reveal any potential differences in growth based on colony size and morphology. (B) DC3000 and CUCPB5506 were also compared for their ability to translocate AvrPto-Cya expressed from plasmid pCPP5702 as indicated by Cya (adenylate cyclase)-dependent increases in cAMP in leaf tissue [11]. CUCPB5113 (DC3000 ΔhrcQ_B_-U::ΩSp^R^/Sm^R^) was used as a T3SS-deficient control [26]. Bacteria were infiltrated into *N. benthamiana* leaves at the indicated CFU/ml. Inoculated leaf tissue was sampled by excision of 0.63-cm diameter leaf discs 7 h post infiltration and processed to determine soluble pmol cAMP µg^−1^ protein, as previously described [11]. Results presented are the mean and standard deviation from three leaves for each treatment.
(4.51 MB TIF)

Figure S2 Growth of the Δ*hopQ1-1*Δ*avrPto*Δ*avrPtoB* mutant in *N. benthamiana* leaves is partially restored by complementation with *avrPtoB*. *N. benthamiana* leaves were infiltrated with the indicated strains at 3×10^4^ CFU/ml (2.5 log CFU/cm^2^ leaf tissue) with a blunt syringe. The avrPtoB gene was expressed from P_avrPto_ in pBBR derivative pCPP5372. Bacterial populations were determined from three 0.8-cm leaf discs 6 days post-inoculation. Results are the mean and standard deviation of bacterial populations collected from four leaf samples. Means marked with the same letter are not statistically different at the 5% confidence level based on Duncan's multiple range test.
(0.20 MB TIF)

Figure S3 Structural alignment of selected HopR1 homologs. The alignment was generated using the MUSCLE algorithm and is displayed in strict CLUSTAL W format.
(0.06 MB TXT)
