# Supplementary figures and images for: Deletions in the Repertoire of Pseudomonas syringae pv. tomato DC3000 Type III Secretion Effector Genes Reveal Functional Overlap among Effectors
Source: PLoS Pathog. 2009 Apr 17;5(4):e1000388. doi: 10.1371/journal.ppat.1000388 (PMC2663052; doi:10.1371/journal.ppat.1000388)

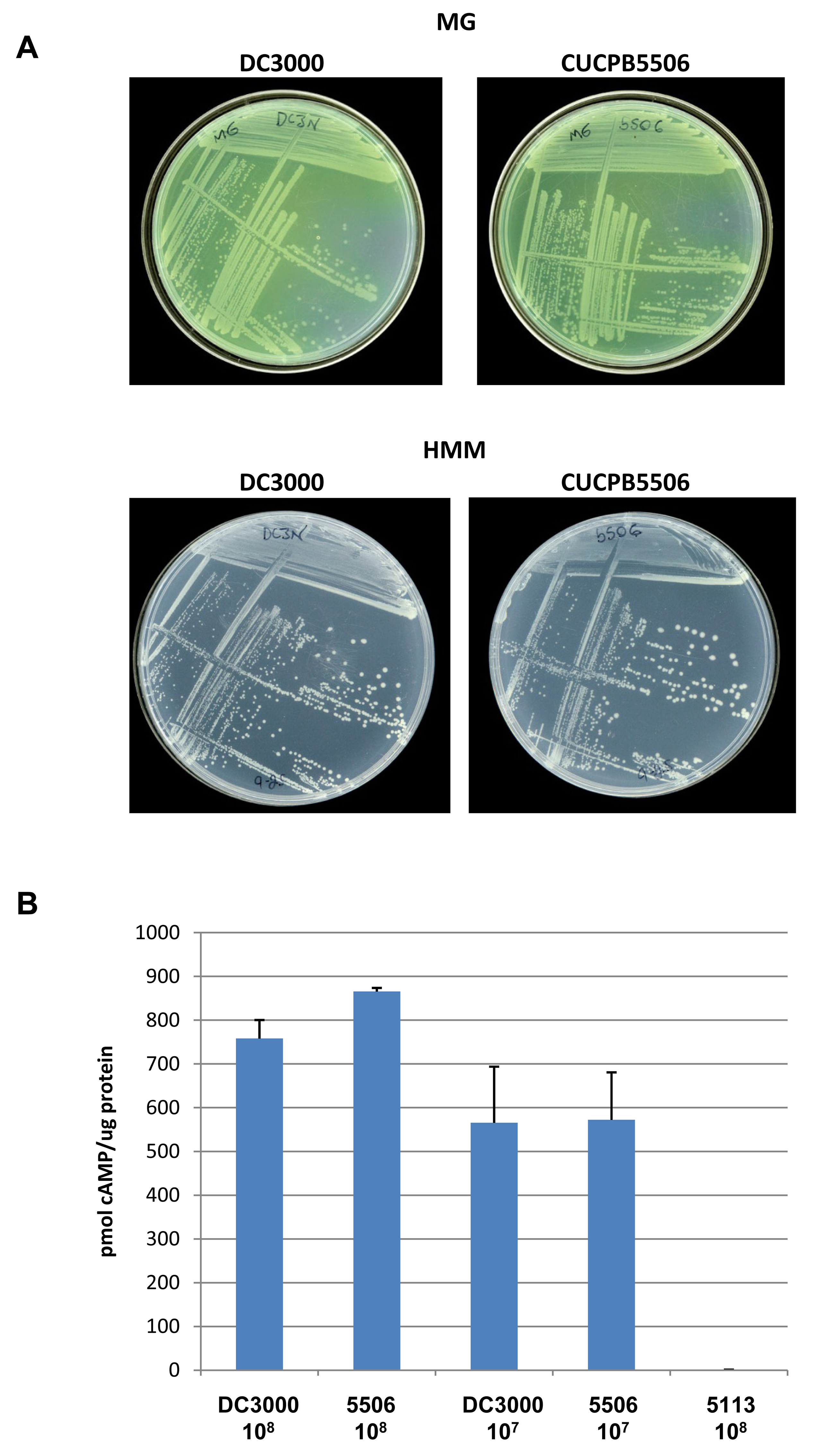

Supplement: Figure S1 — A CUCPB5500 derivative grows on minimal media and translocates AvrPto-Cya as well as wild type DC3000. (A) DC3000 and CUCPB5506 (a CUCPB5500 derivative with the phytotoxin coronatine biosynthesis cfa cluster deleted) were simultaneously streaked on mannitol glutamate (MG) and Hrp minimal medium (HMM) and then photographed 4 days later to reveal any potential differences in growth based on colony size and morphology. (B) DC3000 and CUCPB5506 were also compared for their ability to translocate AvrPto-Cya expressed from plasmid pCPP5702 as indicated by Cya (adenylate cyclase)-dependent increases in cAMP in leaf tissue [11]. CUCPB5113 (DC3000 ΔhrcQB-U::ΩSpR/SmR) was used as a T3SS-deficient control [26]. Bacteria were infiltrated into N. benthamiana leaves at the indicated CFU/ml. Inoculated leaf tissue was sampled by excision of 0.63-cm diameter leaf discs 7 h post infiltration and processed to determine soluble pmol cAMP µg−1 protein, as previously described [11]. Results presented are the mean and standard deviation from three leaves for each treatment. (4.51 MB TIF) [file ppat.1000388.s001.tif]

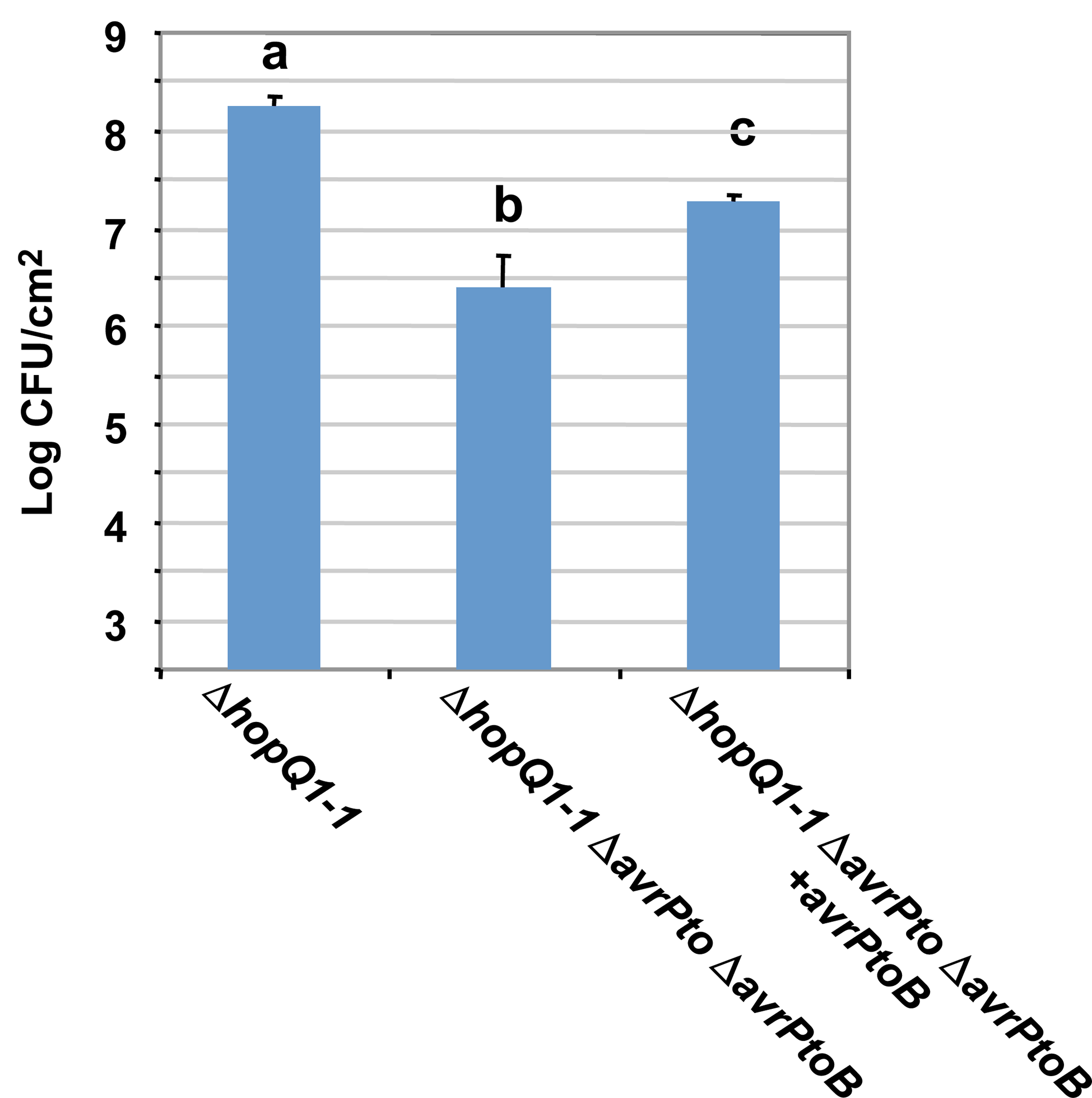

Supplement: Figure S2 — Growth of the ΔhopQ1-1ΔavrPtoΔavrPtoB mutant in N. benthamiana leaves is partially restored by complementation with avrPtoB. N. benthamiana leaves were infiltrated with the indicated strains at 3×10^4^ CFU/ml (2.5 log CFU/cm2 leaf tissue) with a blunt syringe. The avrPtoB gene was expressed from PavrPto in pBBR derivative pCPP5372. Bacterial populations were determined from three 0.8-cm leaf discs 6 days post-inoculation. Results are the mean and standard deviation of bacterial populations collected from four leaf samples. Means marked with the same letter are not statistically different at the 5% confidence level based on Duncan's multiple range test. (0.20 MB TIF) [file ppat.1000388.s002.tif]
